# Effects of Drought Stress on Pollen Sterility, Grain Yield, Abscisic Acid and Protective Enzymes in Two Winter Wheat Cultivars

**DOI:** 10.3389/fpls.2017.01008

**Published:** 2017-06-20

**Authors:** Baodi Dong, Xin Zheng, Haipei Liu, Jason A. Able, Hong Yang, Huan Zhao, Mingming Zhang, Yunzhou Qiao, Yakai Wang, Mengyu Liu

**Affiliations:** ^1^Key Laboratory of Agricultural Water Resources of Chinese Academy of Sciences and Hebei Key Laboratory of Water-Saving Agriculture, Center for Agricultural Resources Research, Institute of Genetics and Developmental Biology, Chinese Academy of SciencesShijiazhuang, China; ^2^School of Agriculture, Food and Wine, Waite Research Institute, The University of Adelaide, AdelaideSA, Australia

**Keywords:** drought stress response, wheat, pollen sterility, antioxidant enzyme, abscisic acid

## Abstract

Drought stress induced pollen sterility is a detrimental factor reducing grain number in wheat. Exploring the mechanisms underlying pollen fertility under drought conditions could assist breeding high-yielding wheat cultivars with stress tolerance. Here, by using two Chinese wheat cultivars subjected to different levels of polyethylene glycol (PEG)-induced drought stress, possible links between pollen fertility and stress tolerance were analyzed under different levels of drought stress at the young microspore stage. In both cultivars, higher grain number reduction was observed under condition of lower water availability. Overall, the drought tolerant cultivar (Jinmai47) exhibited less grain number reduction than the drought sensitive cultivar (Shiluan02-1) under all stress conditions. Compared with Shiluan02-1, Jinmai47 exhibited superior physiological performance in terms of leaf photosynthetic rate, ear carbohydrate accumulation, pollen sink strength, pollen development and fertility under stress. Moreover, Jinmai47 showed a lower increase in endogenous abscisic acid in ears than Shiluan02-1. Furthermore, higher levels of superoxide dismutase (SOD) and peroxidase (POD) activities were also found in the drought tolerant cultivar Jinmai47 under PEG stress, compared with the drought sensitive cultivar Shiluan02-1. Changes in these physiological traits could contribute to better pollen development and male fertility, ultimately leading to the maintenance of grain number under drought stress.

## Introduction

Drought stress is a detrimental abiotic factor that limits crop productivity and quality worldwide (Barnabas et al., 2008; Daryanto et al., 2016). Bread wheat (*Triticum aestivum*) is a major cereal crop grown in north China, where yields have suffered substantially as a consequence of the increasing scarcity of water resources (Zhang and Huang, 2012; Wu et al., 2014). For cereal crops, the extent to which drought stress reduces grain yield largely depends on the developmental stages during which stress is experienced (Dolferus et al., 2011). Drought stress that occurs after anthesis can significantly affect grain filling, thus leading to reduced grain size (Sanjari Pireivatlou and Yazdansepas, 2010), whereas stress that is experienced before or at anthesis mainly affects the grain number (Liu et al., 2015).

Grain number potential in cereals is determined during the early development stage prior to anthesis (Sreenivasulu and Schnurbusch, 2012). Drought stress at this stage significantly affects the development of the floral meristem and can cause a reduction of spikelet initiation and abortion of developing florets (Dolferus et al., 2011). Water-deficit stress during meiosis also affects gametogenesis and reduces the pre-determined grain number (Saini et al., 1984). In self-pollinating crops such as wheat, a critical factor controlling grain number is pollen development during the young microspore stage (YM stage). Previously it has been shown that drought stress and moderate water-deficit stress at this stage of development can dramatically reduce grain number (Saini et al., 1984; Dorion et al., 1996; Ji et al., 2010), which has been linked to pollen sterility during the early reproductive stages (such as YM), whereas ovary fertility remained unaffected (Ji et al., 2010).

Previous studies have shown that the maintenance of tapetum activity and anther sink strength play central roles in well-coordinated pollen development (Clément et al., 1996, 1998; Nguyen et al., 2009; Ji et al., 2010). The tapetum tissue, which provides nutrition for the growing microspores, has been reported to exhibit vigorous metabolism at the YM stage, whereas the anthers showed the highest sink strength in floral organs (Clément et al., 1996). Water-deficit stress causes developmental defects in the tapetum and a lack of starch accumulation in pollen grains (Saini et al., 1984; Nguyen et al., 2009; Ji et al., 2010). In both wheat and rice, it has been reported that male sterility may occur when carbohydrate metabolism is significantly affected (thereby disrupting starch accumulation in the pollen grains) (Dorion et al., 1996; Sheoran and Saini, 1996). However, stress tolerant wheat cultivars can maintain starch accumulation and sink strength during the YM stage under conditions of water stress (Ji et al., 2010). This can be partially explained by the higher expression level of *cell wall invertase* (*TaIVR*) in such cultivars (Koonjul et al., 2005; Ji et al., 2010). *TaIVRs* are expressed in both tapetum and vascular bundle tissues around the anther (Goetz et al., 2001; Koonjul et al., 2005; Oliver et al., 2005), and are key factors controlling carbon assimilate uptake in the tapetum and microspore development (Roitsch, 1999; Sturm and Tang, 1999). Many studies have demonstrated that drought stress can reduce *TaIVR* expression and activity, thereby causing reduced anther sink strength (Dorion et al., 1996; Sheoran and Saini, 1996; Zinselmeier et al., 1999; McLaughlin and Boyer, 2004; Koonjul et al., 2005; Ji et al., 2010). However, the molecular mechanisms regulating TaIVR activity and anther sink strength remain unknown.

As a key phytohormone involved in abiotic stress responses, abscisic acid (ABA) plays a significant role in male fertility during reproductive stress (Singh and Sawhney, 1998; Ji et al., 2011). In cereals, ABA content in the anther is genotype-dependent with stress tolerant genotypes showing lower ABA accumulation under cold and water stress when compared to stress sensitive genotypes (Oliver et al., 2007; Ji et al., 2011). Exogenous ABA applications can reduce TaIVR activity, leading to significant grain loss, whereas the deletion of the key ABA catabolic gene *ABA 8*′*-hydroxylase* (*TaABA8′OH*) has been shown to significantly increase ABA content in the ear (Ji et al., 2011). The importance of ABA in controlling grain number and the maintenance of pollen sink strength possibly relies on its functional roles in sugar signaling (Arenas-Huertero et al., 2000; Laby et al., 2000; Rook et al., 2001) and the regulation of *TaIVR* genes (Oliver et al., 2007; Ji et al., 2011), which are known to affect starch accumulation and pollen fertility (Dorion et al., 1996; Sheoran and Saini, 1996). ABA biosynthesis and catabolism both determine ABA homeostasis in plant tissues (Nambara and Marion-Poll, 2005). Thus, further studies focusing on the mechanisms that control ABA homeostasis, particularly in the ear during reproductive stages would provide useful information for furthering germplasm development.

Although ABA homeostasis affects anther sink strength and male fertility, excess accumulation of reactive oxygen species (ROS) causes oxidative stress and severe cellular damage; subsequently activating stress response pathways affecting plant development (Baxter et al., 2014; You and Chan, 2015). Limited studies on wheat undergoing drought stress during reproductive development have reported oxidative stress adaptation and its effect on yield potential. In rice, *Defective Tapetum Cell Death 1* (*DTC1*) modulates ROS dynamics and controls the degeneration of tapetal cells, ultimately affecting microspore and pollen development (Yi et al., 2015). This suggests that ROS homeostasis and the ability to inactivate excessive ROS also play an important role in male sterility during the reproductive stages.

The early reproductive YM stage is most sensitive to drought stress. However, little is known about the relationships among pollen development at this stage, agronomic characters and photosynthetic characteristics. Moreover, the regulatory mechanisms of wheat cultivars with different drought sensitivity under different drought stress levels need to be further investigated. Therefore, the aims of this study are: (1) to examine the changes in agronomic traits (e.g., grain number) of the drought tolerant and sensitive wheat cultivars under different levels of drought stress during the YM stage; (2) to compare the different responses of photosynthetic characteristics, pollen development and endogenous hormone under drought stress during YM stage; (3) to investigate relevant gene expression in drought tolerant and sensitive cultivars under drought stress; and (4) to compare and analyze the possible factors contributing to the maintenance of grain number and yield under drought stress. To date, there has been limited research regarding drought stress treatments applied to wheat cultivars with different stress sensitivity during the YM stage in the North China Plain. Our results provide a theoretical basis for the identification of drought tolerant wheat cultivars when drought stress is prevalent during reproductive development.

## Materials and Methods

### Wheat Cultivars, Growing Conditions, and Stress Treatments

The wheat cultivars Jinmai47 (semi-winter and mid-early maturity cultivar) and Shiluan02-1 (semi-winter and mid-maturity cultivar) are the main cultivars grown in northern China. Jinmai47 is considered to be a drought tolerant cultivar, whereas Shiluan02-1 is a drought sensitive cultivar (Jin-wen et al., 2013; Xu et al., 2013).

For field experiments, these two wheat cultivars were grown at the Luancheng agro-ecosystem experimental station of Chinese Academy of Sciences under natural conditions. This station is located in Luancheng county of Hebei province (37°53′ N, 114°41′ E, altitude 50.1 m). It is representative of the high wheat production area in the northern part of the North China Plain. The annual average temperature is 12.2°C and the annual precipitation is 530 mm (Dong et al., 2011). Drought stress treatments were achieved using the withholding irrigation method (different irrigation regimes at different wheat growth stages, see **Supplementary Table S1**) with fresh groundwater. The treatment groups (12 in total) were as follows: T0, no irrigation during the whole life cycle; T1a, 60 mm irrigation at the jointing stage; T1b, 240 mm irrigation at the seedling stage before winter; T1c, 240 mm irrigation at the jointing stage; T1d, 240 mm irrigation at grain filling; T2a, 60 mm irrigation at both the jointing and heading stage; T2b, 60 mm irrigation at both the jointing stage and grain filling; T2c, 120 mm irrigation at both the jointing and heading stage; T2d, 120 mm irrigation at both the jointing stage and grain filling; T3a, 60 mm irrigation at the jointing, heading, and grain filling stages; T3b, 80 mm irrigation at the jointing, heading, and grain filling stages; T4, well irrigated across all stages. Randomized block design was used to assign each treatment group with an experimental plot of 288 m^2^ in size (each treatment representing 24 m^2^). The experiments were performed with three biological replicates.

For glasshouse experiments, wheat plants were grown using Hoagland nutrient solution (Hoagland and Arnon, 1950) in pots (25 cm in diameter and 25 cm in height, 15 plants in each pot, five pots for each treatment group) filled with ceramsite. Wheat plants were grown in a controlled growth room chamber under a 16 h (25°C)/8 h (15°C) photoperiod. The YM stage was determined by the auricle distance (AD)-based measurements as described previously (Ji et al., 2010). The AD is the distance between the auricles of the flag leaf and the penultimate leaf. When the AD of the main stem was approximately -2 to 0 cm, the YM stage drought stress was applied using PEG6000 solution in two stress treatment groups (15% PEG6000 for moderate stress; 30% PEG6000 for severe stress) for 5 days, followed by normal nutrient solution treatment. The control group (CK) was treated with normal nutrient solution.

### Measurement of Height, Biomass, Yield and Grain Number

For field experiments, all plants within the 2 m^2^ in each treatment plot were harvested manually and air-dried to determine the aboveground biomass. Plants were threshed using a stationary thresher to obtain the grain yield. The mean value of biomass and grain yield were calculated for each treatment group for both cultivars.

For glasshouse experiments, 20 individual plants in each treatment group were selected (four plants randomly selected per pot, five pots per treatment group) and air-dried. Plant height, the main stem dry weight, ear length, grain number of the main stem ear, and grain yield were measured and the mean value was calculated for each treatment group for both cultivars.

### Anther Microscopic Observation

To study young microspore development and tapetum degeneration under drought stress, anthers from each treatment group were harvested after 1 and 5 days of PEG6000 treatment and then fixed using formaldehyde. Fixed anther samples were washed and dehydrated using ethanol solution followed by xylene de-alcoholisation. The treated samples were embedded in paraffin and 10 μm sections were cut and mounted on poly-Lys-coated glass slides. Wright’s staining (Clark, 1981) was performed and photographs were taken using a Leica TCS-SP8 confocal microscope (Leica Microsystems, Germany). For each treatment group, 15 individual plants (three plants randomly selected per pot, five pots per treatment group) were analyzed.

### Measurement of Photosynthetic and Transpiration Rates

For glasshouse experiments, photosynthetic and transpiration rates were measured in the flag leaf of the main stem for the CK group, 15% PEG treatment group and 30% PEG treatment group. Measurements were taken between 9 – 11 am using a LI-6400XT portable photosynthesis system (LI-COR Biosciences, Lincoln, NE, United States) according to the manufacturer’s protocol. Briefly, when the value of ΔCO_2_ was less than 0.2 μmol/mol, the photosynthetic and transpiration rates were obtained. These measurements were performed with 15 biological replicates (three plants randomly selected per pot, five pots per treatment group).

### Starch Staining and Sugar Measurements

In the glasshouse experiments, starch staining was performed using I_2_- KI solution (0.20% w/w I_2_ and 0.50% w/w KI). For pollen staining, five anthers were harvested from each plant, and pollen was released by gentle pressure in 50 μL NanoPure water. Staining solution was added to the pollen, which was then incubated for 1 min at room temperature. After washing, stained pollen was viewed using a Leica TCS-SP8 confocal microscope (Leica Microsystems, Germany). For ovary staining, ovaries with anthers were obtained from each plant and I_2_- KI staining was performed. Ten individual plants from each treatment group (two plants randomly selected per pot, five pots per treatment group) were analyzed and representative images were shown.

Measurements of soluble sugars in the main stem ear and flag leaf were performed with the anthrone colorimetric method using a Plant Soluble Sugar assay kit (Comin Biotech Co. Ltd, China) according to the manufacturer’s protocol. For each treatment group, five replicates (one plants randomly selected per pot, five pots per treatment group) were harvested and pooled. Measurements of reducing sugars in the main stem ear and flag leaf were performed with 3,5-dinitrosalicylic acid (DNS) reagent using a Reducing Sugar assay kit (Comin Biotech Co. Ltd, China) according to the manufacturer’s protocol.

### RNA Extraction, Reverse Transcription, and Real-Time PCR

For glasshouse experiments, total RNA was extracted from the ear on the main stem of the two wheat cultivars in different treatment groups with three biological replicates using Trizol reagent (Invitrogen, United States). After DNase I (Promega, United States) treatment, total RNA (5 μg for each sample) was reverse transcribed using a FastQuant RT Kit (Tiangen Biotech Co. Ltd, China) according to the manufacturer’s protocol. Real-time PCR analysis was carried out using the Bio-Rad CFX Connect^TM^ Real-Time PCR Detection System (Hercules, United States) with SYBR Green SuperReal PreMix Plus (Tiangen Biotech Co. Ltd, China). PCR conditions were 95°C for 15 min, followed by 40 cycles of 95°C for 10 s and 60°C for 30 s. Three biological replicates were used with three technical replicates. The wheat *ACTIN* gene (GenBank accession No.KC775780) was used as the internal reference. Relative gene expression levels were determined using the 2^-ΔΔCT^ method (Livak and Schmittgen, 2001). The sequences of all the primer are listed in **Supplementary Table S2**.

### ABA Measurement

The main stem ear of five individual plants in each treatment group (one plants randomly selected per pot, five pots per treatment group) was harvested from the 15% PEG treatment group, 30% PEG treatment group and the control group. Fresh weight was measured before each sample was snap frozen and ground in liquid nitrogen. ABA was extracted at 4°C and three technical replicates were performed for each biological replicate. The ABA measurement was carried out in the National Center of Plant Gene Research (Beijing) using a UPLC-MS/MS system (ACQUITYUPLC, United States).

### SOD and POD Activity Measurements

SOD activity in the ear of the main stem was measured with the hydroxylamine method using a Total Superoxide Dismutase (T-SOD) assay kit (Jiancheng Bioengineering Institute, Nanjing, China) according to the manufacturer’s protocol. Main stem ear POD activity measurement was performed using a Peroxidase assay kit (Jiancheng Bioengineering Institute) according to the manufacturer’s protocol. For each treatment group, five samples were harvested (one plants randomly selected per pot, five pots per treatment group) and pooled, before following the assay protocol to obtain enzyme values.

### Statistical Analysis

Statistical analysis was performed using SPSS 22.0 software. Student’s *t*-test was used when comparing gene expression and each trait between Jinmai47 and Shiluan02-1 (such as the biomass and yield of two cultivars in each treatment group in the field experiment). One-way analysis of variance (one-way ANOVA) followed by the Student-Newman–Keuls test (as a *post hoc* test) was used for comparing multiple sets of data within each cultivar (such as comparing the photosynthetic rate of each variety at different time points). All data are presented as mean ± standard error. *P* < 0.05 was considered as significant.

## Results

### Genotypic Differences in Wheat Biomass and Yield Reduction under Drought Stress

**FIGURE 1:**
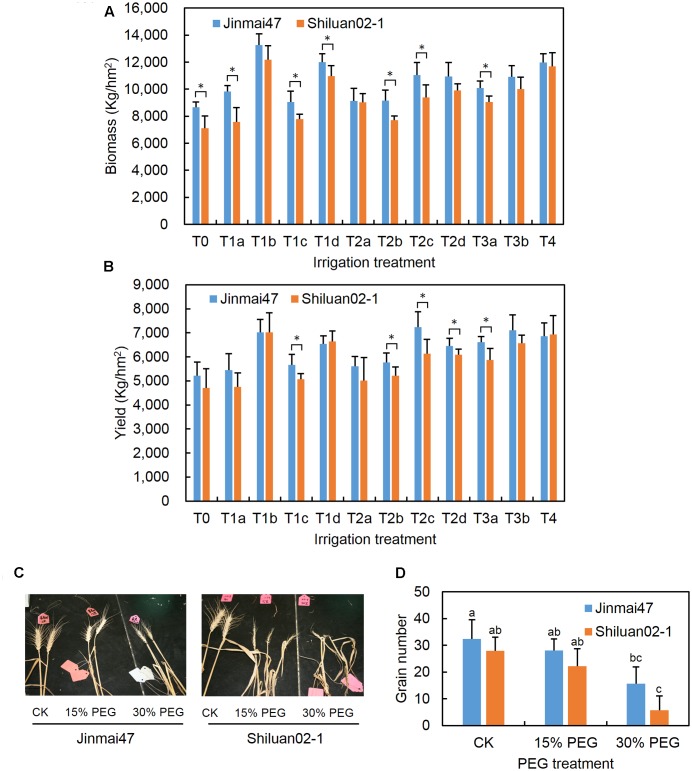
Measurement of wheat biomass, yield, and grain number under drought stress conditions. Jinmai47 and Shiluan02-1 were planted in a field under natural conditions and subjected to the following different irrigation treatments: T0, no irrigation during the entire growth stage; T1a, 60 mm irrigation at the jointing stage; T1b, 240 mm irrigation at the seedling stage before winter; T1c, 240 mm irrigation at the jointing stage; T1d, 240 mm irrigation at grain filling; T2a, 60 mm irrigation at both the jointing and heading stage; T2b, 60 mm irrigation at both the jointing stage and grain filling; T2c, 120 mm irrigation at both the jointing and heading stage; T2d, 120 mm irrigation at both the jointing stage and grain filling; T3a, 60 mm irrigation at the jointing, heading, and grain filling stages; T3b, 80 mm irrigation at the jointing, heading, and grain filling stages; T4, well irrigated across all stages. Biomass **(A)** and yield **(B)** of the two cultivars were measured after harvest. For the glasshouse experiments, wheat plants were treated with 15 or 30% PEG6000 for 5 days at the YM stage, followed by the normal nutrient solution until maturity. After harvest, images of the ears of the two cultivars were recorded **(C)** and main stem ear grain number **(D)** was measured (*n* = 20). CK indicates control group under normal conditions. All values are the means ± standard errors of three independent experiments. Different letters indicate significant differences (Student-Newman–Keuls test, *P* < 0.05) in **(D)**. ^∗^Indicates significant difference (Student’s *t*-test, *P* < 0.05) in **(A,B)**.

The effects of drought stress on the biomass and grain yield of two wheat cultivars with different stress sensitivity were compared under field conditions. As shown in **Figures 1A,B**, both cultivars had improved biomass and yield as irrigation levels were increased. Drought tolerant Jinmai47 appeared to have a significantly higher biomass compared with drought sensitive Shiluan02-1 under T0, T1a, T1c, T1d, T2b, T2c, and T3a irrigation treatments. In the T0 treatment group (no irrigation group) and T1a treatment group (60 mm irrigation at the jointing stage), the mean of the Jinmai47 biomass was 22 and 30% higher than that of Shiluan02-1, respectively. Similarly, drought tolerant Jinmai47 appeared to have a significantly higher yield when compared with Shiluan02-1 across five irrigation treatments (T1c, T2b, T2c, T2d, and T3a). For example, the average yield of Jinmai47 was 12 and 11% higher than that of Shiluan02-1 in T1c and T2b treatment groups, respectively.

### PEG Stress at the YM Stage Reduced Grain Number

To investigate the different responses of the two wheat cultivars during the YM stage, the YM stage of Jinmai47 and Shiluan02-1 was identified using an effective AD based method (Ji et al., 2010). After vernalisation, Jinmai47 and Shiluan02-1 seedlings were transplanted from the field into pots filled with ceramsite. As shown in **Supplementary Figure S1A**, both cultivars showed a transient high sensitivity period at -2 to 2 cm AD. PEG-induced drought stress at this period markedly reduced the grain number. Moreover, Wright’s staining of the microsporangium at -2 cm AD showed that microspores from both cultivars were at the pre-dyad stage (**Supplementary Figure S1B**), suggesting the period is coinciding with the YM stage. Consequently, experiments to simulate drought stress during the YM stage was conducted when plants reached a -2 to 2 cm AD.

Wheat plants with -2 to 2 cm AD were treated with 15% or 30% PEG solution to simulate a moderate or severe drought stress for 5 days, as outlined in the section “Materials and Methods.” Both PEG treatments led to a reduction in main ear grain number in both wheat cultivars (**Figures 1C,D**). The main stem ear grain number of Jinmai47 was reduced by 13.3% under 15% PEG stress when compared with the control. Thirty percent PEG stress caused a 51.8% reduction in the main stem ear grain number of Jinmai47. However, the grain number reduction in Shiluan02-1 was even more pronounced, with reductions of 20.6 and 79.5% for 15 and 30% PEG treatments respectively.

### Pollen Sterility Caused Grain Number Loss

**FIGURE 2:**
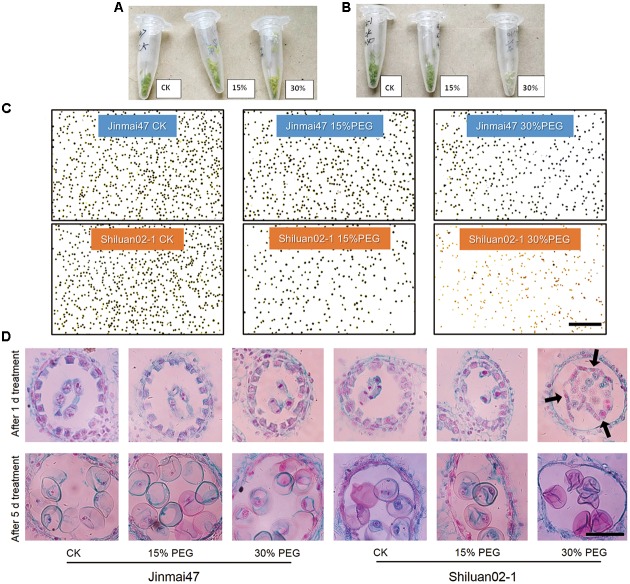
Effect of young microspore (YM) stage polyethylene glycol (PEG) stress on pollen development. Jinmai47 and Shiluan02-1 plants were treated with 15% or 30% PEG6000 for 5 days at the YM stage. Anthers of Jinmai47 **(A)** and Shiluan02-1 **(B)** were harvested and images captured to highlight differences. **(C)** Pollen of the two cultivars was stained with I_2_-KI to determine fertility. Scale bar indicates 0.5 mm. **(D)** Anthers from the two cultivars were harvested after 1 and 5 days of treatment. Wright’s staining of paraffin sections was performed to show YM development and tapetum degeneration. Black arrows indicate shedding of the tapetum. CK indicates control group under normal conditions. Scale bar indicates 0.2 mm.

To investigate the possible mechanisms underlying grain number reduction in the two cultivars under YM drought stress, anther development was investigated. The color of the anther tissue in both Jinmai47 and Shiluan02-1 appeared to change under PEG stress (**Figures 2A,B**). The anther color of Jinmai47 changed from fresh green to yellow–green after being treated with 30% PEG for 5 days. However, the anther color of Shiluan02-1 changed from fresh green to almost white under the 30% PEG stress solution. Further analysis focused on the mature pollen activity evaluated by I_2_-KI staining. As shown in **Figure 2C**, the number of blue-stained pollen grains (fertile pollen) in both cultivars was reduced under PEG stress. However, under the same stress treatment, a greater reduction in fertile pollen was observed in Shiluan02-1. Notably, Shiluan02-1 showed almost no fertile pollen under the 30% PEG stress solution treatment.

Wright’s staining of the microsporangium showed that after 1 day of PEG stress, there was no obvious difference in the tapetum shape between the control group and the PEG treatment groups in Jinmai47 (**Figure 2D**). Interestingly, 1 day of 30% PEG treatment caused a severe developmental defect in Shiluan02-1; whereby the tapetum was separated from the anther wall (**Figure 2D**). After 5 days, programmed degeneration of the tapetum was observed in the control group and PEG treatment groups, while PEG treatments caused a slight acceleration of degeneration in Jinmai47 (**Figure 2D**). Notably, the PEG-induced tapetum defects were more significant in Shiluan02-1, whereas no tapetum was observed in the 30% PEG treatment after 5 days of stress.

### PEG Stress at the YM Stage Reduced Photosynthetic Rate

**FIGURE 3:**
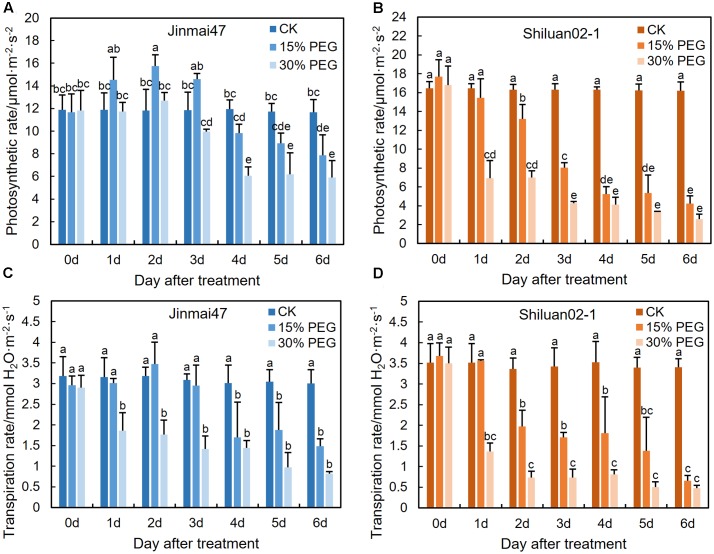
Effect of YM stage PEG stress on photosynthetic rate. Jinmai47 and Shiluan02-1 plants were treated with 15% or 30% PEG6000 for 5 days at the YM stage, followed by normal nutrient solution treatment at 6 days until maturity. Flag leaf photosynthetic rate **(A,B)** and transpiration rate **(C,D)** measurements were performed using a LI-6400XT portable photosynthesis system at 9–11 am each day (*n* = 15). All values are shown as the means ± standard errors. Different letters indicate significant differences (Student-Newman–Keuls test, *P* < 0.05).

To investigate the possible correlations between photosynthetic rate and grain yield under drought stress at the YM stage, photosynthesis and transpiration rates of the flag leaf were measured. The two wheat cultivars exhibited different trends when under stress. In the 15% PEG stress treatment group, the photosynthetic rate of the flag leaf significantly increased in Jinmai47, but decreased significantly in Shiluan02-1 during the first 2 days (**Figures 3A,B**). In contrast, under the 30% PEG stress treatment, both Jinmai47 and Shiluan02-1 showed significant reductions in the photosynthetic rate of the flag leaf, but to a lesser extent in Jinmai47 from 0 to 6 days. The transpiration rate of the two cultivars also exhibited genotype-dependent responses. Compared with CK, the transpiration rate of cultivar Jinmai47 decreased by 46% (15% PEG stress) and 71% (30% PEG stress) on the first re-watering day (6 days after treatment), whereas the transpiration rate of Shiluan02-1 was decreased by 82% (15% PEG stress) and 86% (30% PEG stress) (**Figures 3C,D**), suggesting a significant compensation effect in Jinmai47 when under PEG stress.

PEG stress at the YM stage also affected several agronomic characteristics, such as plant height and main stem dry weight (**Supplementary Figure S2**). The plant height of Jinmai47 was reduced by 31% (15% PEG stress) and 34% (30% PEG stress) when compared with the control at harvest, whereas the plant height of Shiluan02-1 remained unchanged. Similarly, the main stem dry weight of Jinmai47 was reduced by 46% (15% PEG stress) and 58% (30% PEG stress), whereas that of Shiluan02-1 showed no significant change. The ear length of both cultivars appeared to be shortened only marginally under stress when compared with the control.

### PEG Stress at the YM Stage Affected Carbohydrate Metabolism

**FIGURE 4:**
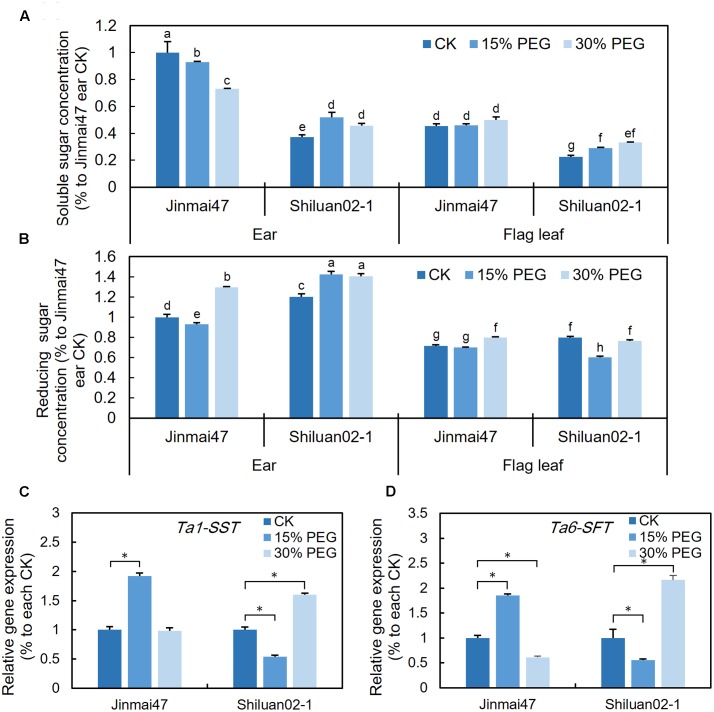
Effect of YM stage PEG stress on carbohydrate accumulation. After 5 days of 15 and 30% PEG6000 treatment, ears and leaves were harvested from Jinmai47 and Shiluan02-1 plants for soluble sugar **(A)** and reducing sugar **(B)** measurements (*n* = 5). Total RNA was extracted from the ears after 5 days treatment, and qPCR expression analysis was performed for *Ta1-SST* **(C)** and *Ta6-SFT* **(D)** (*n* = 3), with normalization to their respective control. All values are shown as the means ± standard errors. Different letters indicate significant differences (Student-Newman–Keuls test, *P* < 0.05) in **(A,B)**. ^∗^Indicates significant difference (Student’s *t*-test, *P* < 0.05) in **(C,D)**.

To investigate whether the altered photosynthetic rate was associated with changes in carbohydrate metabolism, the sugar contents in the wheat ear and flag leaf were measured. For Jinmai47, 5 days of 15% PEG stress slightly reduced the content of either soluble sugars or reducing sugars in the ear (**Figures 4A,B**). However, for Shiluan02-1, the 15% PEG treatment led to a slight increase in both soluble and reducing sugars in both tissues (with the exception of reducing sugars in the flag leaf) (**Figures 4A,B**). Under 30% PEG stress, the soluble sugar content decreased in the ear of Jinmai47, but increased in Shiluan02-1 (**Figure 4A**). In the flag leaf, an increase in soluble sugars was observed only in Shiluan02-1 (**Figure 4A**). For the reducing sugar content, a significant increase could be observed in the 30% PEG treatment when compared with the control in the ear of both cultivars (**Figure 4B**). In the flag leaf, a slight increase of the reducing sugar content under the 30% PEG treatment could only be observed in Jinmai47 (**Figure 4B**).

The qPCR analysis showed that the expression of *Ta1-SST* and *Ta6-SFT* (two fructosan synthesis-related genes) in the Jinmai47 ear increased after 5 days of 15% PEG stress (**Figures 4C,D**). Under 30% PEG stress, compared with the control, no significant increase in the expression of these two genes could be observed in Jinmai47 (**Figures 4C,D**). However, in the drought sensitive Shiluan02-1, *Ta1-SST* and *Ta6-SFT* were down-regulated under 15% PEG stress while under 30% PEG stress both genes were significantly up-regulated (**Figures 4C,D**).

At the YM stage, starch accumulation could be observed in the ovary of the two cultivars, which is consistent with previous report (Ji et al., 2010). Neither PEG treatment appeared to affect starch accumulation in the ovary of the two cultivars (**Supplementary Figure S3**). Interestingly, anther development in both cultivars appeared to be inhibited by the 30% PEG treatment, while developmental defects were observed in the anthers of Shiluan02-1 (**Supplementary Figure S3**).

### PEG Stress at the YM Stage Led to Differences in the Expression of *TaIVR1* and Accumulation of ABA in Wheat Ears

**FIGURE 5:**
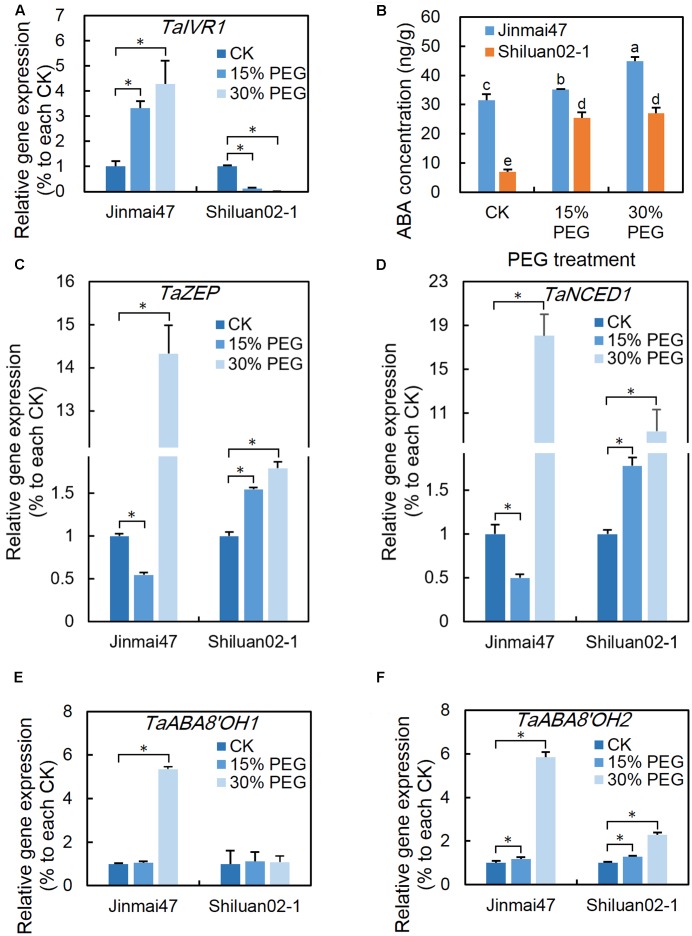
Effect of YM stage PEG stress on *TaIVR1* expression and ABA homeostasis. After 5 days of PEG6000 treatment, ears from Jinmai47 and Shiluan02-1 plants were harvested for gene expression analysis **(A,C–F)** and ABA content **(B)** measurements (*n* = 5). Total RNA was extracted from ears after 5 days of treatment, and qPCR expression analysis was performed for *TaIVR1* **(A)**, *TaZEP* **(C)**, *TaNCED1* **(D)**, *TaABA8*′*OH1* **(E)**, and *TaABA8*′*OH2* **(F)** (*n* = 3) with normalization to their respective control. All values are shown as the means ± standard errors. Different letters indicate significant differences (Student-Newman–Keuls test, *P* < 0.05) in **(B)**. ^∗^Indicates significant difference (Student’s *t*-test, *P* < 0.05) in **(A,C–F)**.

Since *TaIVR1* is very important for grain production in response to drought stress, *TaIVR1* expression level in the ears of both cultivars was analyzed after 5 days of PEG treatment. The qPCR results showed that both the 15 and 30% PEG treatments significantly induced the expression of *TaIVR1* in Jinmai47 (**Figure 5A**). By contrast, the 15% PEG treatment significantly inhibited the expression of *TaIVR1* in Shiluan02-1; with almost no expression of *TaIVR1* detected in Shiluan02-1 under the 30% PEG treatment (**Figure 5A**).

Ear endogenous ABA content in the two wheat cultivars was measured using UPLC-MS/MS. Compared with Shiluan02-1, a higher concentration of ABA was found in the ears of Jinmai47 under control condition (**Figure 5B**). After 5 days of PEG stress at the YM stage, the ABA level in the ears increased significantly in both Jinmai47 and Shiluan02-1, with the ABA level in Jinmai47 increasing by 42% (30% PEG treatment), while the levels in Shiluan02-1 increased almost three times (both 15 and 30% PEG treatments).

Considering that several ABA biosynthesis and catabolism related genes may contribute to the differences in ABA homeostasis, the expression levels of *TaZEP*, *TaNCED1*, *TaABA8*′*OH1*, and *TaABA8*′*OH2* were analyzed. The qPCR results showed that the expression of *TaZEP* (**Figure 5C**) and *TaNCED1* (**Figure 5D**) in Jinmai47 ears was down-regulated under the 15% PEG stress treatment and up-regulated under the 30% PEG stress treatment. In Shiluan02-1, *TaZEP* and *TaNCED1* expression exhibited significant increase under both 15 and 30% PEG stress treatments (**Figure 5D**). In Jinmai47, *TaABA8*′*OH1* increased by 5.3 times and *TaABA8*′*OH2* increased by 5.9 times under the 30% PEG treatment (**Figures 5E,F**, respectively). In Shiluan02-1, 30% PEG stress did not induce significant expression change of *TaABA8*′*OH1* (**Figure 5E**), but caused a 2.3-fold up-regulation of *TaABA8*′*OH2* expression (**Figure 5F**).

### PEG Stress at the YM Stage Affected the Activity of Protective Enzymes in Wheat Ears

**FIGURE 6:**
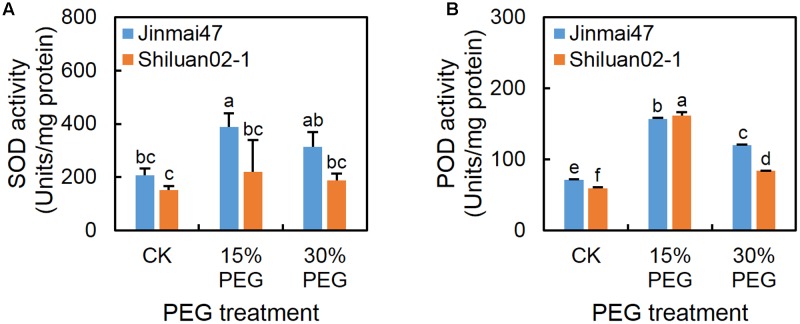
Effect of YM stage PEG stress on antioxidant enzyme activity. Jinmai47 and Shiluan02-1 were grown in the Hoagland nutrient solution in a controlled growth room chamber environment under a 16 h (25°C)/8 h (15°C) photoperiod. After 5 days of PEG6000 treatment, ears from Jinmai47 and Shiluan02-1 plants were harvested for determination of SOD **(A)** and POD **(B)** activity (*n* = 5). All values are shown as the means ± standard errors. Different letters indicate significant differences (Student-Newman–Keuls test, *P* < 0.05).

Under the 15% PEG treatment, the SOD and POD activities increased in both cultivars when compared with the control (except for SOD activity in Shiluan02-1) (**Figure 6**), while the activity of SOD (**Figure 6A**) in Jinmai47 was significantly higher than that in Shiluan02-1. Under the 30% PEG treatment, the POD activity in both cultivars was increased when compared with the control, while lower POD activity was detected in Shiluan02-1 when compared to the Jinmai47 (**Figure 6B**).

## Discussion

High yield stability is one of the key driving objectives for wheat breeding programs in arid and semi-arid areas across the globe. Abiotic stress during early reproductive stages significantly reduces grain number in cereal crops (Dolferus et al., 2011; Fischer, 2011; Liu et al., 2015). Therefore, the study of an early reproductive stage, such as the YM stage, while under stress, is of significance when considering yield improvement. In the present study, the effects of drought stress on drought tolerant and sensitive wheat cultivars were evaluated by investigating reproductive structures, photosynthetic related parameters, protective enzyme levels, ABA level, ABA homeostasis-associated gene expression and agronomic traits. The data presented here highlights the importance of tapetum and pollen development in drought stress adaptation, with insights into the possible physiological mechanisms contributing to the maintenance of reproductive fertility and yield stability in the drought tolerant cultivar.

To date, there have been few studies that have focused on differences between drought tolerant and sensitive wheat cultivars in response to drought stress during the YM stage. Our results confirmed that grain number reduction was more severe with increased drought stress intensity in both drought tolerant and sensitive wheat cultivars, with less reduction in the drought tolerant cultivar (**Figure 1**). In this study, pollen sterility was the main cause of grain number reduction, which is consistent with previous reports (Ji et al., 2010, 2011). After 5 days of 30% PEG stress treatment, dysplastic anthers were observed in the drought sensitive cultivar Shiluan02-1 (**Supplementary Figure S3**). Furthermore, anther color in the Shiluan02-1 cultivar was white and no active pollen grains could be detected by I_2_-KI staining (**Figures 2B,C**). In contrast, the drought tolerant cultivar Jinmai47 had significantly more stained fertile pollen, even under 30% PEG stress (**Figure 2C**). Moreover, observation of the microsporangium in Jinmai47 revealed no obvious differences in microspore and tapetum morphology between the control and PEG groups after 1 day of treatment (**Figure 2D**). However, in Shiluan02-1, clear degeneration and shedding of the tapetum layer was observed after only 1 day of the 30% PEG treatment (**Figure 2D**), which is consistent with a previous report (Nguyen et al., 2009). The tapetal layer is the most active in developing microspores at the YM stage (Barnabas et al., 2008; Dolferus et al., 2011). As tapetum cells can provide nutrients for pollen development (Clément et al., 1996, 1998), the tapetum degeneration observed in this study could be the primary cause of pollen sterility and therefore, grain number loss. In addition, PEG stress did not induce significant changes in ovary development in either wheat cultivar (**Supplementary Figure S3**), which is consistent with a previous report (Ji et al., 2010). These results highlight the importance of a functional tapetum for pollen development during drought stress.

A functional tapetum is also crucial for the maintenance of high sink strength (Dolferus et al., 2011). Assimilate storage ability in the reproductive organs largely depends on pollen sink strength, which is essential for pollen development and germination (Clément et al., 1994; Franchi et al., 1996). Starch accumulation and storage in the pollen of the drought tolerant cultivar Jinmai47 remained unaffected even under 30% PEG stress, while almost no starch storage was observed in the pollen of the drought sensitive cultivar Shiluan02-1 (**Figure 2C**). Low sink strength in Shiluan02-1 could be explained by the premature degeneration of the tapetum mentioned earlier. Moreover, a decrease in starch accumulation in response to PEG stress was associated with the accumulation of soluble and reducing sugars in the ears of the two wheat cultivars. Under 15% PEG stress, soluble and reducing sugar contents in the drought tolerant Jinmai47 ears only exhibited a slight reduction (**Figures 4A,B**), indicating a continuing supply for starch accumulation. In contrast, both 15 and 30% PEG stress treatments led to an increase in the soluble and reducing sugar contents in ears of the drought sensitive cultivar Shiluan02-1 (**Figures 4A,B**). This may be attributable to a repression of TaIVR activity and inhibition of starch biosynthesis. TaIVRs regulate starch biosynthesis and play a central role in the maintenance of sink strength (Tiessen et al., 2002; Roitsch and González, 2004), and decreased TaIVR activity could lead to a decrease in the starch content of anther tissues (Dorion et al., 1996; Sheoran and Saini, 1996). In this study, *TaIVR1* expression in ears was analyzed with significantly induced expression detected in the drought tolerant cultivar after PEG treatment (**Figure 5A**), indicating the strong feedback signaling and a relatively higher TaIVR activity. In the drought sensitive cultivar Shiluan02-1, a sharp reduction in *TaIVR1* expression was observed after 5 days of PEG treatment (**Figure 5A**), which may imply reduced TaIVR activity when compared with Jinmai47. Given that the drought sensitive wheat cultivar showed lower *TaIVR* expression levels during stress (Ji et al., 2010), lower TaIVR activity may be the main attribute leading to reduced starch accumulation. Further analysis of TaIVR activity will provide more information on the mechanisms regulating starch accumulation and pollen sink strength under reproductive drought stress.

Fructan accumulation under abiotic stress might also contribute to the maintenance of photosynthesis and stress adaptation (Pilon-Smits et al., 1995; Hincha et al., 2002; Pollock et al., 2003). In this study, the expression of the fructan biosynthesis related genes *Ta1-SST* and *Ta6-SFT* was up-regulated in the drought tolerant cultivar in response to the 15% PEG stress treatment (**Figures 4C,D**), indicating a possible increase in fructan biosynthesis. However, both *Ta1-SST* and *Ta6-SFT* were down-regulated in the drought sensitive cultivar in response to 15% PEG stress (**Figures 4C,D**), suggesting lower fructan production. Accordingly, the photosynthetic and transpiration rates of Jinmai47 increased during the first 3 days of 15% PEG stress and then decreased, while those parameters of Shiluan02-1 decreased at all times during stress (**Figure 3**). Under 30% PEG stress, the expression of both *Ta1-SST* and *Ta6-SFT* was significantly increased in Shiluan02-1, which was in contrast to the response observed in Jinmai47 (*Ta1-SST* remained unchanged while *Ta6-SFT* decreased) (**Figures 4C,D**). This may be explained by carbon assimilation starvation in Shiluan02-1, as both the photosynthetic and transpiration rates of Shiluan02-1 were significantly lower than those in Jinmai47 under 30% PEG stress (**Figures 4C,D**).

As a central regulator of abiotic stress response in plants, ABA is involved in regulating sugar accumulation and sink strength (Oliver et al., 2005, 2007; Ji et al., 2011). Exogenous ABA treatment caused repressed *TaIVR1* expression and pollen sterility in wheat (Ji et al., 2011), which suggests that excessive accumulation of ABA in anthers affects carbohydrate metabolism and pollen development. In this study, ABA content in the ears increased in both drought tolerant and sensitive cultivars under the PEG treatment. A higher ABA increase was observed in Shiluan02-1 under PEG stress, although the actual ABA content was still higher in Jinmai47 under PEG stress (**Figure 5B**). As 30% PEG stress significantly affected the sink strength of Shiluan02-1 but not Jinmai47, these results indicate that the regulation of endogenous ABA level is important for pollen development. Similar results have been reported previously (Oliver et al., 2007; Ji et al., 2011), and further exogenous ABA treatment experiments would presumably provide more evidence for ABA-regulated pollen development in the wheat cultivars studied.

Genotype-dependent differences in the ABA content of the two wheat cultivars may be a consequence of the differential regulation of ABA metabolism-related genes under stress. A previous study has demonstrated that a low ABA level in wheat is correlated with low ABA biosynthesis-related gene expression and high ABA catabolism-related gene expression (Ji et al., 2011). In the present study, 15% PEG stress did not strongly affect the expression of the ABA catabolism-related gene *TaABA8*′*OH1* in either cultivar. However, ABA biosynthesis-related genes *TaZEP* and *TaNCED1* were down-regulated in Jinmai47, but up-regulated in Shiluan02-1 (**Figure 5**). This result may explain the higher accumulation of ABA content in Shiluan02-1. Under 30% PEG stress, the expression of *TaABA8*′*OH1* and *TaABA8*′*OH2* was significantly induced in Jinmai47, whereas only a slight increase of *TaABA8*′*OH2* expression was observed in Shiluan02-1 (**Figure 5**), indicating the stronger regulatory capacity in the drought tolerant cultivar under 30% PEG stress. The higher SOD and POD activities during the YM stage under PEG stress observed in Jinmai47 are another possible attribute to drought stress tolerance. Under abiotic stress conditions, overproduction of ROS triggers the activation of scavenging mechanisms (You and Chan, 2015), which is consistent with the observation made in the present study. Although under 15% PEG stress, SOD and POD activities were increased in the ears of both cultivars (except for SOD activity in Shiluan02-1), the drought tolerant cultivar Jinmai47 exhibited a higher increase in SOD level (**Figure 6**). Under 30% PEG stress, POD activity in the ear of Jinmai47 was also considerably higher than that in Shiluan02-1. Studies characterizing the link between ROS metabolism, protective enzyme activity and pollen development, especially at the molecular level, would further our understanding in this area.

**FIGURE 7:**
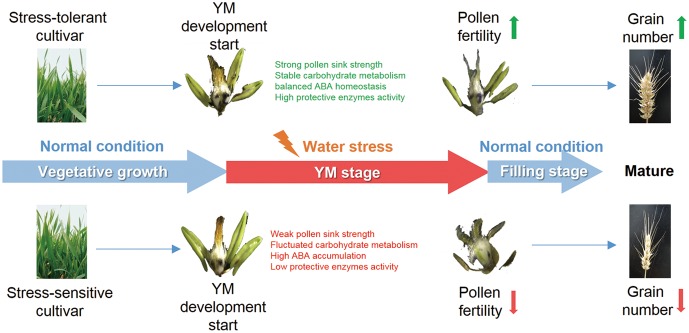
Proposed model for how drought affects grain number at the YM stage in wheat. Both drought tolerant and sensitive cultivars suffer from YM stage drought stress. However, the drought tolerant cultivar shows a stronger sink strength and better pollen development, which may be a consequence of stable carbohydrate metabolism, balanced ABA homeostasis, and high protective enzyme activities. Such changes could lead to enhanced pollen fertility and higher grain number in the drought tolerant cultivar.

In summary, this research has demonstrated the effects of YM stage drought stress on pollen fertility and grain yield in two wheat cultivars, and investigated the physiological parameters that can affect reproductive fertility. Compared with the drought sensitive cultivar, the tolerant cultivar exhibited better pollen sink strength, stable carbon assimilation, higher protective enzyme activities, coordinated carbohydrate metabolism and ABA content. These physiological traits could potentially contribute to better pollen development and male fertility, ultimately leading to the maintenance of grain number under drought stress (**Figure 7**). The results of this study provide insight into the stress response mechanisms during drought stress at early reproductive stages in wheat. Further study of the molecular regulatory pathways controlling pollen fertility will contribute to an elucidation of the genetic basis of drought tolerance in wheat.

## Additional Information

Accession codes: Sequences of the genes in this paper may be found in the GenBank database library under the following accession numbers: KC775780 (*TaACTIN*), AB159786.1 (*Ta1-SST*), FJ228688.1 (*Ta6-SFT*), AF030420.1 (*TaIVR1*), AK332872 (*TaZEP*), CA731387 (*TaNCED1*), CN011303 (*TaABA8*′*OH1*), CD919420 (*TaABA8*′*OH2*).

## Author Contributions

BD designed and performed the experiments, and prepared the manuscript. XZ performed gene expression analysis and prepared the manuscript. HL and JA modified the manuscript. HY performed anther microscopic observation. HZ performed starch staining. MZ and YQ measured photosynthetic and transpiration rate. YW performed sugar measurement. ML supervised this project and prepared the manuscript.

## Conflict of Interest Statement

The authors declare that the research was conducted in the absence of any commercial or financial relationships that could be construed as a potential conflict of interest.

## Supplementary Material

FIGURE S1 Effects of PEG stress occurring at different developmental stages on grain number. **(A)** The AD of the main stem of Jinmai47 and Shiluan02-1 plants was measured. Plants with different ADs were exposed to a 30% PEG6000 treatment for 5 days, followed by the normal nutrient solution treatment until maturity. Grain number on the main stem was measured at harvest. A total of 34 and 53 main stems of Jinmai47 and Shiluan02-1 (respectively) were analyzed. Both cultivars exhibited the highest sensitivity of grain number to water stress at the -2 to 2 cm AD stage. **(B)** Anthers from the two cultivars were harvested at the -2 cm AD stage. Wright’s staining of paraffin sections was performed to demonstrate the development of the young microspore at this stage. Scale bar indicates 0.2 mm.

FIGURE S2 Effects of YM stage PEG stress on agronomic traits. Jinmai47 and Shiluan02-1 plants were treated with either 15% or 30% PEG6000 for 5 days at the YM stage, followed by normal nutrient solution treatment until maturity. At harvest, plant height **(A)**, main stem dry weight **(B)**, and main ear length **(C)** were measured.

FIGURE S3 Effects of YM stage PEG stress on starch accumulation in the ovary. Jinmai47 and Shiluan02-1 plants were treated with either 15% or 30% PEG6000 for 5 days at the YM stage. Ovaries with anthers were obtained and I_2_- KI staining was performed. Ten individual plants from each treatment group were analyzed and representative images are shown. The scale bar indicates 1 mm.

TABLE S1 Different irrigation regimes at different wheat growth stages used in the field experiments.

TABLE S2 Sequences of the primers used in this study.
