# Supplementary figures and images for: Effects of Drought Stress on Pollen Sterility, Grain Yield, Abscisic Acid and Protective Enzymes in Two Winter Wheat Cultivars
Source: Front Plant Sci. 2017 Jun 20;8:1008. doi: 10.3389/fpls.2017.01008 (PMC5476748; doi:10.3389/fpls.2017.01008)

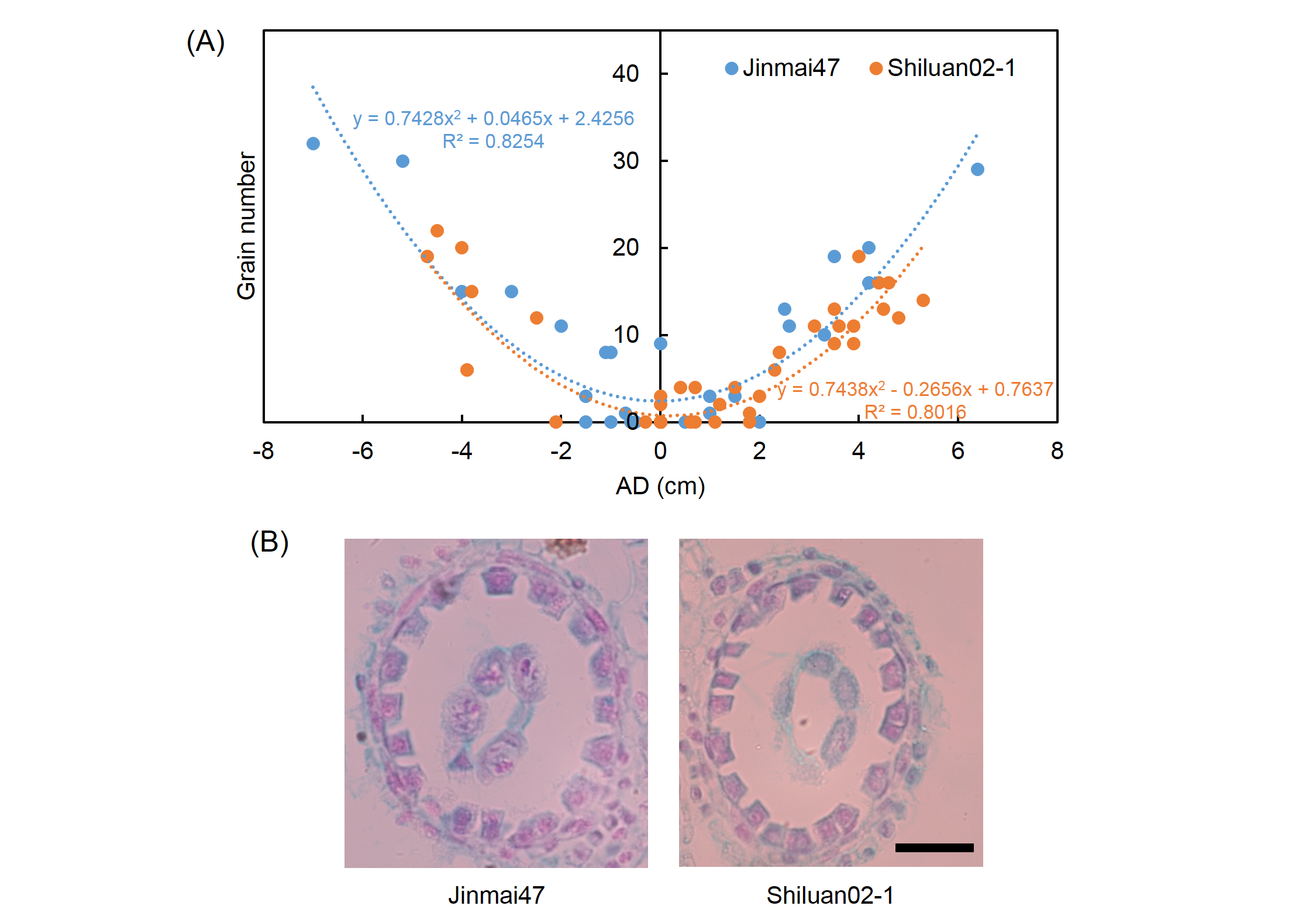

Supplement: FIGURE S1 — Effects of PEG stress occurring at different developmental stages on grain number. (A) The AD of the main stem of Jinmai47 and Shiluan02-1 plants was measured. Plants with different ADs were exposed to a 30% PEG6000 treatment for 5 days, followed by the normal nutrient solution treatment until maturity. Grain number on the main stem was measured at harvest. A total of 34 and 53 main stems of Jinmai47 and Shiluan02-1 (respectively) were analyzed. Both cultivars exhibited the highest sensitivity of grain number to water stress at the -2 to 2 cm AD stage. (B) Anthers from the two cultivars were harvested at the -2 cm AD stage. Wright’s staining of paraffin sections was performed to demonstrate the development of the young microspore at this stage. Scale bar indicates 0.2 mm. [file Image_1.TIF]

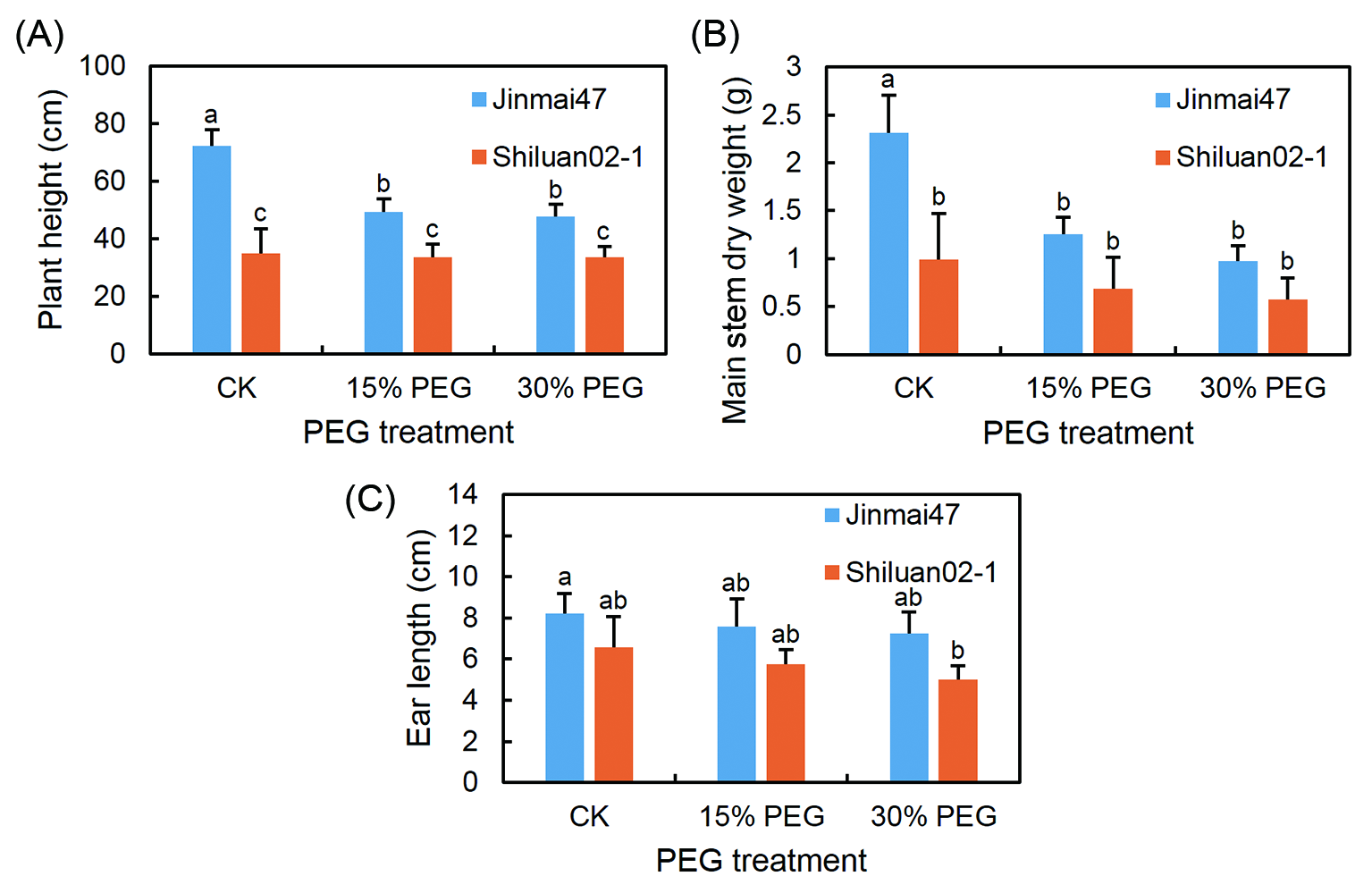

Supplement: FIGURE S2 — Effects of YM stage PEG stress on agronomic traits. Jinmai47 and Shiluan02-1 plants were treated with either 15% or 30% PEG6000 for 5 days at the YM stage, followed by normal nutrient solution treatment until maturity. At harvest, plant height (A), main stem dry weight (B), and main ear length (C) were measured. [file Image_2.TIF]

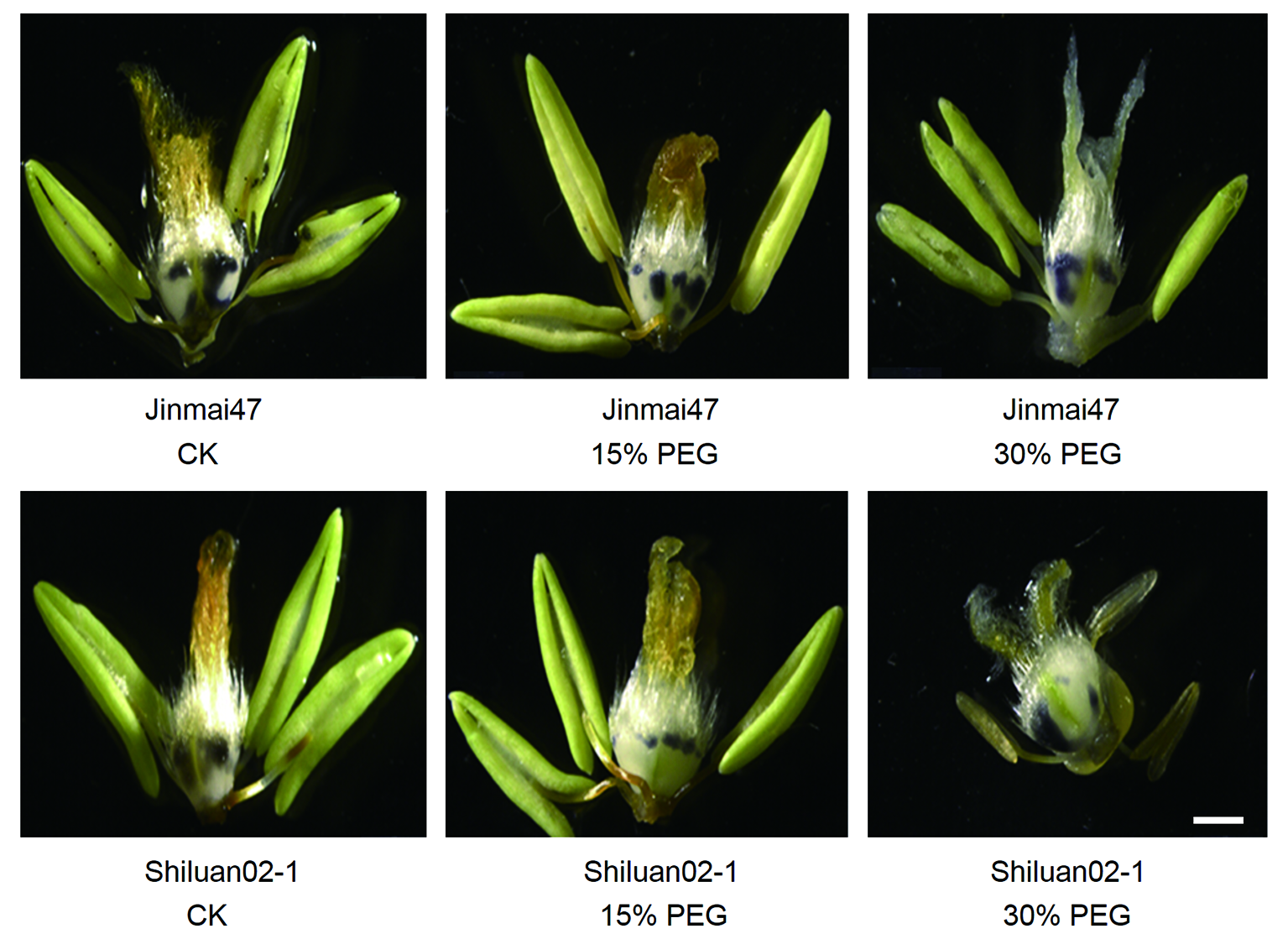

Supplement: FIGURE S3 — Effects of YM stage PEG stress on starch accumulation in the ovary. Jinmai47 and Shiluan02-1 plants were treated with either 15% or 30% PEG6000 for 5 days at the YM stage. Ovaries with anthers were obtained and I2- KI staining was performed. Ten individual plants from each treatment group were analyzed and representative images are shown. The scale bar indicates 1 mm. [file Image_3.TIF]
