# Supplementary material for: Effects of Drought Stress on Pollen Sterility, Grain Yield, Abscisic Acid and Protective Enzymes in Two Winter Wheat Cultivars
Source: Front Plant Sci. 2017 Jun 20;8:1008. doi: 10.3389/fpls.2017.01008 (PMC5476748; doi:10.3389/fpls.2017.01008)
Supplement: TABLE S1 — Different irrigation regimes at different wheat growth stages used in the field experiments. [file Table_1.DOCX]

**Table S1.** **Different irrigation regimes at different wheat growth stages used in the field experiments.**

| Treatment group | Amount of irrigation at different stages | | | |
| --- | --- | --- | --- | --- |
|  | **Seedling stage before winter** | **Jointing stage** | **Heading stage** | **Grain filling stage** |
| T0 | - | - | - | - |
| T1a | - | 60 mm | - | - |
| T1b | 240 mm | - | - | - |
| T1c | - | 240 mm | - | - |
| T1d | - | - | - | 240 mm |
| T2a | - | 60 mm | 60 mm | - |
| T2b | - | 60 mm | - | 60 mm |
| T2c | - | 120 mm | 120 mm | - |
| T2d | - | 120 mm | - | 120 mm |
| T3a | - | 60 mm | 60 mm | 60 mm |
| T3b | - | 80 mm | 80 mm | 80 mm |
| T4 | 60 mm | 60 mm | 60 mm | 60 mm |
