# Supplementary material for: Effects of Drought Stress on Pollen Sterility, Grain Yield, Abscisic Acid and Protective Enzymes in Two Winter Wheat Cultivars
Source: Front Plant Sci. 2017 Jun 20;8:1008. doi: 10.3389/fpls.2017.01008 (PMC5476748; doi:10.3389/fpls.2017.01008)
Supplement: TABLE S2 — Sequences of the primers used in this study. [file Table_2.DOCX]

**Table S2.** **Sequences of the primers used in this study.**

| Name | Sequence (5'-3') |
| --- | --- |
| Ta1SST-F | AGCGACTCTGCCTATCACTTC |
| Ta1SST-R | GCCATTTTCATTCCCCAAC |
| Ta6SFT-F | AGGCCTATCAGGAGGCAAAAG |
| Ta6SFT-R | CGAGTRATCGTCCATATTGG |
| TaZEP-F | GGAGTTATGAGAAGGAGAGAAAGC |
| TaZEP-R | AAAACGACAAAGGTCCCAGA |
| TaNCED-F | CCTCGAAGCCCAGCACTAAT |
| TaNCED-R | GAGAGCGAGAGGTCCAATGG |
| TaABA8'OH1-F | ACAGATGGTCCACCTCCAAG |
| TaABA8'OH1-R | CCTCTATCGTGCCGTTGATT |
| TaABA8'OH2-F | GGTGATTTTGGAGAGCCTGA |
| TaABA8'OH2-R | AAGTAGTCCGGGCTGTGATG |
| TaIVR1-F | ACGAGGGTCTACCCGAGGAA |
| TaIVR1-R | TACTGCCCCAGACGCTTGTT |
| TaActin-F | CCTCTCTGCGCCAATCGT |
| TaActin-R | TCAGCCGAGCGGGAAATTGT |
